# Gene–Diet Interactions in High-Density Lipoprotein Cholesterol-Related Polymorphisms and Cardiovascular Disease Risk: Insights from the Korean Genome and Epidemiology Study

**DOI:** 10.3390/nu17050778

**Published:** 2025-02-24

**Authors:** Jong-Hee Lee, Kyung-Won Hong, Byoung-Jin Park, Ja-Eun Choi, Dong-Hyuk Jung

**Affiliations:** 1 Department of Family Medicine, Yongin Severance Hospital, Yonsei University College of Medicine, Yongin 16995, Republic of Korea; jongheel@yuhs.ac (J.-H.L.); bjpark96@yuhs.ac (B.-J.P.); 2 Institute of Advanced Technology, THERAGEN Health Co., Ltd., Seongnam-si 13493, Republic of Korea; kyungwon.hong@theragenhealth.com (K.-W.H.); jaeun.choi@theragenhealth.com (J.-E.C.)

**Keywords:** ischemic stroke, cardiovascular disease, HDL cholesterol, GWAS, nutrition

## Abstract

**Background**: Understanding gene–diet interactions is crucial for establishing dietary guidelines for cardiovascular diseases (CVD). This study analyzed the interaction between dietary intake and six genome-wide association study (GWAS)-identified single nucleotide polymorphisms (SNP) associated with high-density lipoprotein (HDL) cholesterol levels and their impact on CVD risk. **Methods**: A total of 68,806 participants in the Korean Genome and Epidemiology Study (KoGES) were analyzed. Six target SNPs (LPL: rs17482753; ABCA1: rs1883025; APOA5: rs651821; LIPC: rs1077835; CETP: rs17231506; and LIPG: rs9953437) were extracted from genome-wide SNP genotype data. Dietary intake was assessed using a food frequency questionnaire. SNP genotyping was conducted using the Korea Biobank Array (Korean Chip), a specialized genotyping platform designed for GWAS of blood biochemical traits in the Korean population. SNP–diet interactions on CVD risk were analyzed using generalized linear models (GLM). **Results**: Among the six SNPs, ABCA1: rs1883025 and APOA5: rs651821 showed significant gene–diet interactions. For rs1883025 (ABCA1), carriers of the T allele exhibited reduced HDL cholesterol levels. However, in the high-protein intake group, individuals with the T/T genotype had a significantly lower risk of ischemic stroke compared to those in the low-protein intake group (interaction *p*-value = 0.044). For rs651821 (APOA5), carriers of the T allele also had lower HDL cholesterol levels, but individuals with the C/C genotype (wild-type homozygotes) in the low-fat intake group showed a significantly reduced risk of coronary artery disease (interaction *p*-value = 0.0155). **Conclusions**: This study suggests potential interactions between polymorphisms associated with low HDL cholesterol and dietary patterns, particularly high-protein and low-fat diets, in relation to CVD risk. These findings highlight the importance of personalized dietary recommendations based on genetic profiles to reduce CVD risk. They provide a basis for future research aimed at developing precision nutrition guidelines and targeted interventions to manage hypo-HDL cholesterolemia and nutrition-related CVD risks.

## 1. Introduction

Ischemic stroke, accounting for 87% of all strokes, remains a leading cause of global morbidity and mortality [1]. Despite advances in treatment, it continues to impose significant public health burdens due to long-term neurological disabilities. Dyslipidemia is a key modifiable risk factor for ischemic stroke, primarily through its contribution to atherosclerosis [2]. Low HDL cholesterol levels have been strongly associated with increased ischemic stroke risk and worsened long-term stroke outcomes, such as stroke recurrence and post-stroke major adverse cardiovascular events [3]. Recently, very high HDL cholesterol levels were also associated with higher risks of all-cause mortality, cardiovascular mortality, and stroke [4]. These findings collectively emphasize the importance of maintaining optimal HDL cholesterol levels for stroke prevention, especially in high-risk populations. As previous studies highlight cardiovascular disease (CVD) risks at both extremes of HDL cholesterol levels, this study aimed to consider both low and high HDL cholesterol levels in relation to dietary factors and CVD risk.

HDL cholesterol levels and function are influenced by genetic predispositions and lifestyle factors, including diet [5,6]. Genetic studies have identified multiple loci associated with HDL cholesterol regulation and vascular disease risk, including LPL, CETP, LIPC, APOA5, and ABCA1 [7]. Particularly, genes related to lipid metabolism that affect stroke risk, including LPL, CETP, and APOA5, have also been identified by previous studies [8,9]. Dietary factors are critical modulators of lipid metabolism and HDL cholesterol function [10,11,12]. Diets rich in unsaturated fats improve HDL cholesterol functionality and reduce CVD risk, while high-protein diets have been associated with favorable metabolic profiles, such as enhanced insulin sensitivity and improved body composition. Emerging evidence suggests that the type of protein consumed (animal vs. plant-based) may differentially affect lipid metabolism and HDL functionality, with plant-based proteins generally being more beneficial for cardiometabolic health. This is particularly relevant in the context of ischemic stroke, as both insulin resistance and altered lipid metabolism are significant contributors to stroke risk and recurrence. Given the distinct dietary patterns in Korean populations, which include higher consumption of plant-based proteins like legumes, soy products, and fermented foods, it is crucial to explore how these protein sources interact with genetic factors. However, studies examining macronutrient-specific effects and their interactions with genetic factors remain limited, underscoring the need for further investigation in diverse populations with distinct dietary patterns, such as Asians.

This study aims to investigate the interaction between HDL-associated genetic variants and macronutrient intake in a large Korean cohort with respect to ischemic stroke risk. Specifically, we hypothesize that HDL cholesterol levels mediate the relationship between macronutrient intake and cardiovascular risk and that genetic variants associated with HDL metabolism interact with dietary patterns to influence cardiovascular outcomes. By analyzing the effects of carbohydrates, proteins, and fats on HDL cholesterol levels and their subsequent impact on ischemic stroke risk, our study provides novel insights into gene–diet interactions and contributes to the development of precision nutrition strategies for reducing ischemic stroke risk and improving cardiovascular health.

## 2. Materials and Methods

### 2.1. Study Population

**Figure 1:**
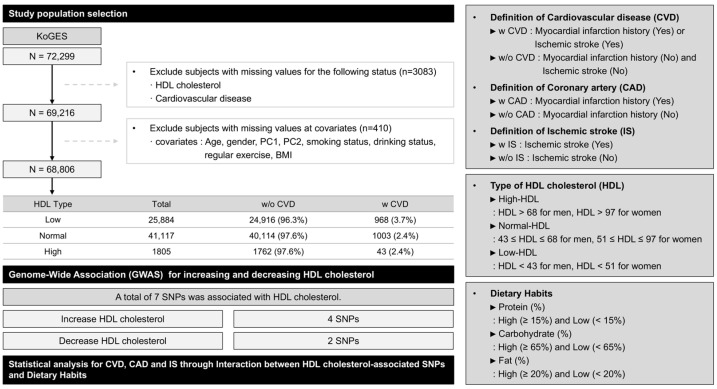
Study design and population characteristics.

Participants for this investigation were drawn from the Korean Genome and Epidemiology Study (KoGES) cohort, an ongoing genome-based epidemiological study conducted by the National Institute of Health, Korea Disease Control and Prevention Agency. The study included 72,299 samples, with clinical, epidemiological, and genomic data obtained through the agency’s official distribution procedures. All participants provided informed consent as part of the KoGES project, and the study protocol was approved by the Institutional Review Board (IRB) of Yong-In Severance Hospital (IRB No: 2020-0040-002). The flowchart of the study population is shown in Figure 1. The population represents the general Korean population, encompassing individuals with a broad range of dietary habits, reflecting typical dietary variability within this population.

### 2.2. Measurement of Anthropometric and Biochemical Parameters

Data collection encompassed questionnaires assessing participants’ medical history, medication use, family history, and dietary habits, along with standardized clinical measurements. Clinical data included comprehensive health check-up results and large-scale SNP genotyping data generated from a Korean-specific SNP microarray provided by domestic genetic testing institutions. Smoking status was categorized as current, former, or non-smoker, while alcohol consumption was classified as current, former, or non-drinker. Regular exercise was defined as participation in sports activities that induced sweating on a regular basis.

Anthropometric and clinical measurements were conducted following standardized protocols. BMI was calculated using participants’ weight and height to assess obesity. Blood pressure measurements were performed twice using a standard mercury sphygmomanometer, with the average values used for analysis. Blood samples were collected after overnight fasting. All lipid measurements, including total cholesterol, HDL cholesterol, and triglycerides, were conducted using enzymatic methods in a certified clinical laboratory following standardized protocols. Quality control procedures were implemented to ensure the accuracy and reliability of the results. These procedures included daily calibration of the assay equipment using standard reference materials and regular participation in an external quality assurance program. Duplicate measurements were performed on randomly selected samples to monitor precision, and the coefficient of variation (CV) for lipid measurements was maintained within acceptable limits. Cardiovascular disease status, encompassing coronary artery disease, ischemic heart disease, and myocardial infarction, was determined based on self-reported questionnaires administered by well-trained examiners during follow-up visits. When a participant reported an incident CVD event in the medical history questionnaire, in-depth personal interviews were conducted to confirm the reported diagnosis. To improve the reliability of the survey, interviewers ensured that participants had received their diagnosis from a healthcare professional.

### 2.3. Definition of Nutrition Intake

To assess the dietary intake patterns of participants, the KoGES project used a semi-quantitative food frequency questionnaire (FFQ) comprising 103 food items. The food composition table used in the nutrient intake calculation was the seventh edition Food Composition Table of Korea [13]. The FFQ is recognized as a useful tool for examining relationships between dietary habits and chronic diseases in large population-based studies and has been validated in a previous study [13]. Participants reported their frequency and portion size of food consumption over the past year, as the FFQ was designed to capture habitual dietary intake over a 12-month recall period. While self-reported dietary data may introduce recall bias and misreporting, the FFQ used in KoGES has been validated against 12-day dietary records and biomarkers, demonstrating reasonable reproducibility and validity. To enhance data reliability, nutrient intake amounts were calculated based on these responses and categorized into high- and low-consumption groups according to the 2020 Korean Dietary Reference Intakes (DRIs). This study focused on macronutrient intake, including carbohydrates, proteins, and fats. The intake categories for each macronutrient were based on the Acceptable Macronutrient Distribution Ranges (AMDR) provided by the Korean Ministry of Food and Drug Safety. According to the AMDR, the recommended proportions of macronutrients in total energy intake (TEI) were carbohydrates 55–65%, proteins 7–20%, and fats 15–30%. For the analysis, macronutrient intake was further classified into three categories: carbohydrates: high ≥ 65% and low < 65%; protein: high ≥ 15% and low < 15%; fat: high ≥ 20% and low < 20%.

### 2.4. Genotype Analysis

Fasting blood samples were collected in serum separator tubes and two ethylenediaminetetraacetic acid (EDTA) tubes. DNA extracted from these blood samples was sent to the National Biobank of Korea for further analysis. SNP genotyping was conducted using the Korea Biobank Array (Korean Chip), a specialized genotyping platform designed for GWAS of blood biochemical traits in the Korean population. The Korean Chip contains over 833,000 markers, including more than 247,000 rare or functional variants identified through sequencing of over 2500 Koreans. Detailed information about the Korean Chip is available in a previous publication [14]. To ensure high-quality genotyping results, strict quality control (QC) criteria were applied. SNPs with a call rate below 97%, a missing genotype rate exceeding 0.01, a minor allele frequency (MAF) less than 0.01, and a Hardy–Weinberg equilibrium *p*-value below 0.000001 were excluded. After QC, the experimental genotypes were phased using ShapeIT v2 and IMPUTE v2 was used for imputation analysis of the phased genotype data with 1000 Genomes Phase3 data as a reference panel [14]. After imputation, imputed variants with an imputation quality score < 0.4 or MAF < 1% were excluded from further analysis. (PMID 30718733) Finally, the number of SNPs for the GWAS was 7,975,321 SNPs from chromosomes 1 to 22 [14].

### 2.5. Statistical Analysis

For statistical analysis, genotypes were extracted from six SNPs known to be strongly associated with HDL cholesterol: LPL (rs17482753), ABCA1 (rs1883025), APOA5 (rs651821), LIPC (rs1077835), CETP (rs17231506), and LIPG (rs9953437). SNP–diet interactions on CVD risk were analyzed using generalized linear models (GLM) under an additive genetic model, adjusting for age, gender, principal components (PC1 and PC2), BMI, smoking status, drinking status, regular exercise, and total energy intake (kcal). All statistical analyses were performed using PLINK (v1.9), with a threshold of *p* < 0.05 for both logistic regression and GLM analyses.

## 3. Results

### 3.1. Baseline Characteristics of the Study Population

As shown in Table 1, the study population (*n* = 68,806) was stratified into three groups based on HDL cholesterol levels: Low-HDL (*n* = 25,884), Normal-HDL (*n* = 41,117), and High-HDL (*n* = 1805). The mean age was 54.09 ± 8.33 years, with the Normal-HDL group being slightly younger than the Low- and High-HDL groups. Females constituted 64.23% of the total population, with the highest proportion in the Low-HDL group (68.1%) and the lowest in the High-HDL group (12.9%). The prevalence of cardiovascular disease, hypertension, type 2 diabetes, and dyslipidemia was significantly higher in the Low-HDL group compared to the Normal- and High-HDL groups (all *p* < 0.001).

Lifestyle factors differed significantly among groups, with current drinking and smoking more common in the High-HDL group, which also showed the highest prevalence of regular exercise. Regarding anthropometric measurements, body mass index and waist circumference were highest in the Low-HDL group and lowest in the High-HDL group (all *p* < 0.001). Blood pressure showed a different pattern, being highest in the High-HDL group and lowest in the Normal-HDL group (all *p* < 0.001). Among biochemical markers, HbA1c, hsCRP, insulin, and triglyceride levels were highest in the Low-HDL group, while the High-HDL group showed the lowest mean levels of HbA1c, insulin, and triglycerides.

### 3.2. Identifying HDL Cholesterol Associated SNPs

Through our GWAS analysis, we selected a total of 6 HDL cholesterol-associated SNPs. The frequently reported lead SNPs for each locus are described in Table 2. Among the 6 identified loci, four SNPs—rs17482753 (*LPL* gene), rs1077835 (*LIPC* gene), rs17231506 (*CETP* gene), and rs9953437 (*LIPG* gene)—were associated with a reduced risk of low HDL and an increased likelihood of high HDL, suggesting these genetic variants may confer protective effects against low HDL cholesterol levels. Conversely, two SNPs—rs1883025 (*ABCA1* gene) and rs651821 (*APOA5* gene)—were associated with an increased risk of low HDL and decreased likelihood of high HDL, indicating their potential role in predisposition to low HDL cholesterol levels.

### 3.3. HDL Cholesterol SNP and Macronutrients Interaction

Table 3 summarizes the interaction *p*-values between six HDL cholesterol-related SNPs and dietary habits on CVD outcomes. Among these, only two SNPs showed significant interactions: ABCA1 SNP with protein intake (interaction *p*-value = 0.044) and APOA5 SNP with fat intake (interaction *p*-value = 0.0155).

For the ABCA1 (rs1883025) SNP, while the T allele was associated with reduced HDL cholesterol levels (as shown in Table 2), individuals with the T/T genotype in the high-protein intake group exhibited a significantly lower risk of ischemic stroke compared to those in the low-protein intake group (Table 3).

Similarly, for the APOA5 (rs651821) SNP, the T allele was associated with reduced HDL cholesterol levels (Table 2). However, in the low-fat intake group, individuals with the C/C genotype (wild-type homozygotes, non-T allele carriers) showed a significantly lower risk of coronary artery disease (CAD) (Table 3).

The remaining SNPs (LPL: rs17482753; LIPC: rs1077835; CETP: rs17231506; and LIPG: rs9953437) showed no statistically significant interactions with dietary habits in relation to HDL cholesterol levels or CVD risk.

**Figure 2:**
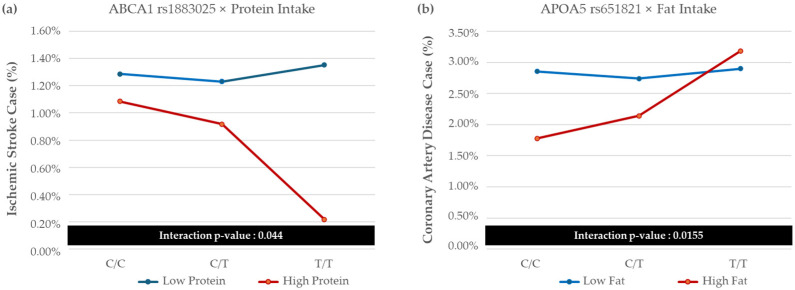
Plots showing the relationship between gene and diet interaction. (**a**) Interaction of ABCA1 rs1883025 with protein intake on ischemic stroke risk. (**b**) Interaction of APOA5 rs651821 with fat intake on coronary artery disease risk.

Figure 2 shows the relationships between gene–diet interactions: (A) the interaction between ABCA1 rs1883025 and protein intake on ischemic stroke risk, and (B) the interaction between APOA5 rs651821 and fat intake on coronary artery disease risk.

## 4. Discussion

We identified significant gene–diet interactions influencing ischemic stroke and CAD risk in individuals with extreme HDL cholesterol levels. The most notable finding was the interaction between the ABCA1 rs1883025 variant and dietary protein intake, where T allele carriers exhibited a reduced risk of ischemic stroke despite their low HDL cholesterol phenotype when their protein consumption was high. Additionally, high-fat diets exacerbated CAD risk in individuals carrying the T allele of the APOA5 rs651821 variant, which is also associated with low HDL cholesterol levels. While odds ratios (ORs) less than 1 suggest a protective effect against low HDL cholesterol, the practical significance of relatively small effect sizes (e.g., OR = 0.747) must be interpreted in the context of population-wide genetic and dietary influences. Although small OR deviations from 1 may seem modest, they can still have meaningful implications in large-scale genetic epidemiology studies, where cumulative effects across populations and environmental exposures contribute to overall cardiovascular risk. Additionally, the observed effect sizes may be amplified when considered in conjunction with specific dietary habits, as demonstrated in our gene–diet interaction analysis. Our findings underscore the potential role of dietary components in modulating genetic susceptibility to cardio-cerebrovascular diseases through HDL cholesterol metabolism.

The relevance of these genetic associations across diverse populations has been an area of growing interest. The SNPs analyzed in this study, including rs1883025 (ABCA1) and rs651821 (APOA5), have been associated with HDL cholesterol levels and cardiovascular risk in multiple populations, including European, East Asian, and South Asian cohorts. Genome-wide association studies (GWAS) have consistently identified these variants as key modulators of lipid metabolism, though their effect sizes vary across ethnic groups, likely due to differences in genetic background, environmental factors, and dietary patterns. Despite their known role in dyslipidemia, the interaction between these SNPs and specific macronutrient intake has been underexplored, particularly in Asian populations. Given the distinct dietary habits and metabolic responses observed in East Asians compared to Western populations, our findings highlight the importance of considering ethnic-specific gene–diet interactions in cardiovascular disease risk assessment. Future studies should investigate whether these interactions hold in other populations, particularly in Western cohorts with differing dietary compositions, to determine whether these effects are generalizable or population specific.

The protective effect of high-protein diets in ABCA1 rs1883025 T allele carriers likely involves the modulation of ABCA1 gene-mediated lipid metabolism. ABCA1 plays a pivotal role in reverse cholesterol transport (RCT), facilitating cholesterol efflux to lipid-poor apolipoproteins such as apoA-I, which contribute to HDL cholesterol biogenesis and functionality [15]. Variants in ABCA1 have been associated with both higher and lower HDL cholesterol levels, depending on the polymorphism [16,17]. However, rs1883025 (T allele) has been linked to reduced cholesterol efflux efficiency, leading to lower circulating HDL cholesterol levels. High dietary protein intake may enhance AMPK and PPAR signaling, which can upregulate ABCA1 expression, thereby partially compensating for the genetic impairment and improving cholesterol transport efficiency. This compensatory mechanism may explain the observed reduction in ischemic stroke risk in T allele carriers despite their lower HDL levels. Additionally, dietary protein may support hepatic lipid homeostasis, reducing lipid accumulation and systemic inflammation, which are key factors in atherosclerosis progression.

Similarly, the APOA5 gene plays a critical role in triglyceride metabolism by enhancing lipoprotein lipase (LPL) activity, which promotes the clearance of triglyceride-rich lipoproteins [18]. APOA5 variants such as rs66s799, rs2075291, and p.S19W have been associated with higher triglyceride and lower HDL cholesterol levels, contributing to arterial stiffness and increased CAD risk [19,20]. This risk is particularly exacerbated under high-fat dietary conditions in genetically susceptible individuals, where excessive fat intake can impair triglyceride clearance by downregulating LPL activity and suppressing PPARα expression, a key regulator of lipid metabolism. This metabolic imbalance leads to increased circulating triglyceride levels, HDL remodeling, and systemic inflammation, ultimately contributing to endothelial dysfunction and CAD progression. Our findings suggest that high-fat diets may exacerbate CAD risk in APOA5 rs651821 carriers by overwhelming the lipid-clearing capacity of LPL, leading to triglyceride accumulation, impaired HDL metabolism, and chronic inflammatory responses. These observations provide new mechanistic insights into gene–diet interactions affecting cardiovascular disease risk, emphasizing the importance of dietary modification in genetically susceptible individuals.

SNPs with OR > 1 indicate an increased genetic predisposition to low HDL cholesterol levels, which is mechanistically linked to impaired lipid metabolism. For example, rs1883025 in ABCA1 is associated with reduced cholesterol efflux capacity due to its role in reverse cholesterol transport (RCT). The ABCA1 transporter is critical for mediating cholesterol efflux from peripheral tissues to nascent HDL particles, a process essential for maintaining HDL function. A genetic variant that disrupts this process leads to lower circulating HDL levels and impairs the anti-atherogenic properties of HDL cholesterol. Similarly, rs651821 in APOA5 is involved in triglyceride metabolism, where its dysfunction leads to an accumulation of triglyceride-rich lipoproteins, which inversely correlates with HDL cholesterol levels. APOA5 is a key regulator of triglyceride hydrolysis via lipoprotein lipase (LPL), and a reduction in its function results in inefficient triglyceride clearance and secondary HDL remodeling. This contributes to hypo-HDL cholesterolemia and increased cardiovascular risk, particularly in the presence of high-fat dietary intake. These mechanisms further explain why gene–diet interactions play a crucial role in modulating lipid metabolism and cardiovascular disease risk.

Emerging evidence further demonstrates the interplay between genetic risk and diet quality in lipid metabolism. For instance, in the Malmö Diet and Cancer cohort, individuals with high genetic risk scores for dyslipidemia showed increased sensitivity to poor-quality diets low in fruits, vegetables, and unsaturated fats [21]. Similarly, Kim et al. found that men with high genetic risk scores for dyslipidemia were more susceptible to hyperlipidemia when consuming instant noodles and soft drinks, while genetically susceptible women were more affected by coffee consumption [22]. Conversely, Park et al. demonstrated that diets rich in whole grains and soybean products significantly reduced dyslipidemia risk in individuals with high genetic risk scores [23]. These studies collectively highlight the importance of dietary modification in individuals with genetic susceptibility to dyslipidemia and cardiovascular outcomes.

Although the roles of ABCA1 and APOA5 in lipid regulation are well-documented, their specific interactions with dietary components in modulating cardio-cerebrovascular disease risk remain underexplored. While some studies suggest dietary macronutrients influence lipid metabolism through pathways such as AMPK, PPAR, and mTOR signaling, the extent to which genetic variants modulate these responses in relation to dietary changes remains unclear. Further research utilizing functional analyses, such as transcriptomic, proteomic, and metabolomic profiling, is needed to determine how dietary intake influences gene expression and lipid regulatory mechanisms in individuals with genetic susceptibility to dyslipidemia.

From a clinical and nutritional perspective, our findings have significant implications for precision nutrition strategies aimed at preventing cardio-cerebrovascular diseases. Traditionally, dietary guidelines have provided generalized recommendations, but our study supports a more individualized approach, where dietary macronutrient intake can be tailored to genetic risk profiles. For instance, ABCA1 rs1883025 T allele carriers may benefit from a high-protein diet to mitigate ischemic stroke risk, while APOA5 rs651821 carriers should be cautious about high-fat intake due to its potential to exacerbate CAD risk. Such genetically guided dietary interventions could enhance cardiovascular disease prevention strategies by optimizing diet based on an individual’s genetic profile.

Dietary protein may influence ABCA1 activity through pathways such as mTOR and AMPK signaling, which regulate gene expression, lipid flux, and energy homeostasis [24]. Additionally, dietary protein may indirectly enhance cholesterol efflux by modulating peroxisome proliferator-activated receptors (PPARs) and liver X receptors, which are key upstream regulators of ABCA1 transcription [25]. These pathways also contribute to improved metabolic profiles, including enhanced insulin sensitivity and reduced inflammation, further supporting HDL cholesterol’s anti-inflammatory and antioxidative properties [26]. Collectively, these mechanisms provide a plausible biological basis for the protective effect of high-protein diets in T allele carriers of the ABCA1 rs1883025 variant. Similarly, high-fat diets, particularly those rich in saturated fats, increase CAD risk in genetically susceptible individuals by elevating circulating triglyceride levels, which may overwhelm the lipid-clearing capacity of LPL [27]. Prolonged consumption of high-fat diets may downregulate PPARα activity, a key regulator of APOA5 expression and LPL activation, further exacerbating triglyceride accumulation and the buildup of lipid intermediates that disrupt metabolic pathways [28]. Additionally, impaired triglyceride metabolism stimulates the secretion of pro-inflammatory cytokines, such as TNF-α, IL-6, and IL-1β, leading to systemic inflammation and endothelial dysfunction—both of which accelerate the progression of atherosclerosis [29]. These pathways offer a plausible explanation for how the APOA5 rs651821 variant, characterized by reduced APOA5 function, synergistically interacts with high-fat diets to amplify CAD risk.

This study uniquely demonstrates the interaction between dietary protein and HDL-associated genetic variants in modulating cardio-cerebrovascular diseases risk. The large sample size and comprehensive genetic analysis enhance the robustness of these findings. By examining macronutrient-specific effects, particularly those of dietary protein and fat, this study addresses critical gaps in understanding how genetic predispositions and dietary factors influence HDL cholesterol metabolism and cardio-cerebrovascular outcomes. Given the distinct genetic and dietary patterns of the Korean population compared to Western populations, these findings contribute significantly to the growing evidence on population-specific gene–diet interactions. Furthermore, they underscore the potential for precision nutrition strategies tailored to regional and genetic contexts for effective prevention of cardio-cerebrovascular diseases.

However, this study has several limitations. One important limitation is the relatively small sample size of the High-HDL group (N = 1805), which may introduce potential sampling bias and limit the reliability of certain estimates. In particular, the low prevalence of conditions such as cardiovascular disease and hypertension in this group may be influenced by the small sample size, making additional subgroup analyses challenging. This may also affect the generalizability of findings related to the High-HDL category. Future studies with larger sample sizes will be necessary to further validate our findings and provide more robust conclusions regarding the relationship between HDL levels, genetic factors, and health outcomes.

As our study is cross-sectional, causal relationships between dietary intake, genetic variants, and disease risk cannot be inferred. Longitudinal cohort studies or randomized controlled trials are necessary to establish cause-and-effect relationships and further validate these gene–diet interactions. Self-reported dietary data may introduce recall bias. In addition, our study did not adjust for other potential confounders such as medication use, socio-economic status, environmental influences, or influence of other dietary habits which may have impacted both dietary intake and cardiovascular outcomes. Future studies should incorporate these variables to reduce potential biases and strengthen the validity of gene–diet interactions. Furthermore, the lack of differentiation between protein (e.g., animal vs. plant protein) and fat (e.g., saturated vs. unsaturated fat) sources reduces specificity, as these sources may exert varying effects on lipid metabolism. Given that different protein and fat sources have been shown to influence lipid metabolism in varying ways, future research should incorporate detailed dietary composition analyses to refine nutritional recommendations for individuals with genetic susceptibility to dyslipidemia.

In this study, macronutrient intake was categorized into high and low groups based on predefined thresholds to facilitate statistical interpretation of gene–diet interactions. While this approach provides a clear distinction between groups, analyzing macronutrient intake as a continuous variable could offer deeper insights into dose–response relationships and the dynamic nature of gene–diet interactions. Future research should investigate whether incremental increases or decreases in macronutrient intake modulate genetic effects on HDL metabolism and cardiovascular risk, as this could enhance our understanding of the interplay between dietary patterns and genetic predisposition.

Additionally, by focusing primarily on common genetic variants, such as the ABCA1 rs1883025 variant, this study did not account for rare large-effect variants, which may substantially contribute to HDL cholesterol extremes. Prior studies, including those by Dron et al., have highlighted the polygenic nature of HDL cholesterol extremes, involving contributions from both rare large-effect and common small-effect variants [30]. Considering other genetic factors beyond the selected SNPs by conducting whole-genome sequencing and rare variant association analyses could help identify additional genetic contributors to HDL metabolism and cardio-cerebrovascular disease risk. Incorporating polygenic risk scores that include both common and rare variants may allow for a more comprehensive genetic risk assessment. Furthermore, this study did not assess HDL functionality, such as cholesterol efflux capacity, which emerging evidence suggests may be a better predictor of cardiovascular outcomes than HDL cholesterol levels alone [31]. As cholesterol efflux capacity reflects the ability of HDL particles to remove excess cholesterol from macrophages, a key process in atheroprotection, future studies should incorporate functional assessments of HDL metabolism to better understand the mechanistic role of HDL in cardio-cerebrovascular disease prevention.

## 5. Conclusions

In conclusion, this study highlights significant interactions between dietary protein and fat intake and HDL-associated genetic variants, particularly the ABCA1 rs1883025 and APOA5 rs651821 variants, in modulating ischemic stroke and CAD risk, respectively. These findings provide novel evidence for gene–diet interactions influencing HDL cholesterol metabolism and ischemic stroke, emphasizing the potential for genetically informed dietary interventions. Future studies should incorporate longitudinal analyses, functional investigations of ABCA1 and APOA5 activity, and differentiation between protein and fat sources to refine precision nutrition strategies for cardio-cerebrovascular health.

## Figures and Tables

**Table 1:** Baseline characteristics of the study population according to HDL Cholesterol.

	Total Population	Low-HDL Group	Normal-HDL Group	High-HDL Group	*p*-Value
N	68,806	25,884	41,117	1805	
Age (years)	54.09 ± 8.33	54.95 ± 8.4	53.49 ± 8.21	55.38 ± 8.56	<0.001
Female (%)	44,191 (64.23)	17,616 (68.1)	26,342 (38.28)	233 (12.9)	<0.001
Cardiovascular disease (*n*, %)	66,792 (97.07)/ 2014 (2.93)	24,916 (96.26)/ 968 (3.74)	40,114 (97.56)/ 1003 (2.44)	1762 (97.62)/ 43 (2.38)	<0.001
Hypertension (*n*, %)	54,792 (79.64)/ 14,011 (20.36)	19,595 (75.7)/ 6289 (24.3)	33,734 (82.05)/ 7380 (17.95)	1463 (81.05)/ 342 (18.95)	<0.001
Type 2 diabetes (*n*, %)	63,994 (93.03)/ 4796 (6.97)	23,509 (90.85)/ 2369 (9.15)	38,794 (94.37)/ 2313 (5.63)	1691 (93.68)/ 114 (6.32)	<0.001
Dyslipidemia (*n*, %)	62,014 (90.15)/ 6777 (9.85)	23,231 (89.76)/ 2650 (10.24)	37,135 (90.34)/ 3970 (9.66)	1648 (91.3)/ 157 (8.7)	0.012
Lifestyle					
Drinking status: Never/Quit/Current (*n*, %)	35,316 (51.33)/ 2880 (4.19)/ 30,610 (44.49)	15,225 (58.82)/ 1279 (4.94)/ 9380 (36.24)	19,812 (48.18)/ 1519 (3.69)/ 19,786 (48.12)	279 (15.46)/ 82 (4.54)/ 1444 (80.0)	<0.001
Smoking status: Never/Quit/Current (*n*, %)	49,528 (71.98)/ 10,976 (15.95)/ 8302 (12.07)	19,191 (74.14)/ 3527 (13.63)/ 3166 (12.23)	29,678 (72.18)/ 6709 (16.32)/ 4730 (11.5)	659 (36.51)/ 740 (41.0)/ 406 (22.49)	<0.001
Exercise status: No/Yes (*n*, %)	33,627 (48.87)/ 35,179 (51.13)	13,646 (52.72)/ 12,238 (47.28)	19,270 (46.87)/ 21,847 (53.13)	711 (39.39)/ 1094 (60.61)	<0.001
Anthropometric traits					
Body mass index (kg/m^2^)	23.99 ± 2.91	24.7 ± 2.87	23.61 ± 2.86	22.59 ± 2.69	<0.001
Waist circumference (cm)	81.19 ± 8.7	83.09 ± 8.39	80.05 ± 8.72	79.73 ± 8.01	<0.001
Systolic blood pressure (mmHg)	122.14 ± 15.34	122.8 ± 15.49	121.63 ± 15.23	124.2 ± 14.95	<0.001
Diastolic blood pressure (mmHg)	75.86 ± 9.98	76.21 ± 10.02	75.57 ± 9.95	77.37 ± 9.83	<0.001
Biochemical traits					
Fasting plasma glucose (mg/dL)	95.18 ± 19.98	96.79 ± 21.89	94.1 ± 18.59	96.98 ± 20.48	<0.001
HbA1c	5.72 ± 0.75	5.85 ± 0.85	5.66 ± 0.69	5.62 ± 0.7	<0.001
hs-CRP	0.26 ± 1.28	0.38 ± 1.74	0.19 ± 0.9	0.19 ± 0.74	<0.001
Insulin	7.61 ± 4.59	8.12 ± 4.83	7.18 ± 4.34	5.98 ± 2.89	<0.001
Total cholesterol (mg/dL)	197.45 ± 35.67	190.91 ± 35.93	201.06 ± 34.89	209.02 ± 35.19	<0.001
HDL cholesterol (mg/dL)	52.81 ± 13.11	41.04 ± 5.66	58.98 ± 10.05	80.75 ± 12.4	<0.001
Triglyceride (mg/dL)	129.02 ± 88.79	165.4 ± 109.78	107.86 ± 64.25	88.65 ± 52.11	<0.001
r-glutamyltransferase	30.69 ± 41.18	29.37 ± 33.76	30.5 ± 42.5	53.58 ± 80.62	<0.001
AST	24.11 ± 21.62	24.32 ± 30.37	23.82 ± 13.32	27.94 ± 23.37	<0.001
ALT	22.73 ± 22.59	24.07 ± 27.41	21.81 ± 17.61	24.47 ± 39.64	<0.001
ALP	163.24 ± 105.59	162.85 ± 109.05	163.91 ± 96.03	153.97 ± 206.1	<0.001
Albumin	4.57 ± 0.47	4.5 ± 0.6	4.6 ± 0.38	4.68 ± 0.3	<0.001
Blood urea nitrogen	14.56 ± 4.06	14.4 ± 4.25	14.62 ± 3.91	15.54 ± 4.21	<0.001
Creatinine	0.82 ± 0.21	0.83 ± 0.23	0.81 ± 0.2	0.91 ± 0.27	<0.001
Uric acid	4.71 ± 1.28	4.82 ± 1.31	4.61 ± 1.26	5.18 ± 1.26	<0.001

*p*-values were calculated using one-way ANOVA or Pearson’s chi-square test.

**Table 2:** Lead SNPs for the low and/or high groups GWASs.

SNP ID	Chr	BP	Minor Allele	Nearby Gene	Normal vs. Low HDL		Normal vs. High HDL	
					OR (95% CI)	*p*-Values	OR (95% CI)	*p*-Values
rs17482753	8	19832646	T	*LPL*	0.747 (0.721–0.774)	3.331 × 10^−58^	1.374 (1.252–1.507)	1.954 × 10^−11^
rs1883025	9	107664301	T	*ABCA1*	1.177 (1.147–1.208)	8.152 × 10^−35^	0.761 (0.698–0.829)	4.459 × 10^−10^
rs651821	11	116662579	C	*APOA5*	1.472 (1.436–1.509)	2.531 × 10^−206^	0.763 (0.702–0.829)	1.831 × 10^−10^
rs1077835	15	58723426	G	*LIPC*	0.781 (0.763–0.799)	7.007 × 10^−98^	1.367 (1.275–1.466)	1.364 × 10^−18^
rs17231506	16	56994528	T	*CETP*	0.591 (0.572–0.61)	4.364 × 10^−226^	1.937 (1.789–2.098)	1.872 × 10^−59^
rs9953437	18	47120600	A	*LIPG*	0.848 (0.829–0.868)	1.287 × 10^−45^	1.226 (1.144–1.315)	8.809 × 10^−9^

Abbreviations. SNP, single nucleotide polymorphism; Chr, chromosome; BP, base pairs; OR, odds ratio; CI, confidence interval. Note: *p*-values were calculated through a genome-wide association study (GWAS) comparing Normal-HDL and Low-HDL groups using PLINK version 1.9.0, adjusted for age, gender, principal components 1 and 2 (PC1 and PC2), smoking status, drinking status, regular exercise, and BMI.

**Table 3:** Statistical analysis results of interactions between HDL cholesterol-associated SNPs and dietary habits.

SNP ID	Mapped Gene	Target Disease	Interaction *p*-Value ^a^ by SNP × Macronutrients		
			Protein ^b^	Carbohydrate ^c^	Fat ^d^
rs17482753	*LPL*	CAD	0.54	0.99	0.26
		IS	0.30	0.10	0.12
		CVD	0.22	0.38	0.09
rs1883025	*ABCA1*	CAD	0.51	0.37	0.51
		IS	0.04	0.95	0.75
		CVD	0.11	0.62	0.77
rs651821	*APOA5*	CAD	0.58	0.12	0.02
		IS	0.94	0.76	0.81
		CVD	0.51	0.22	0.05
rs1077835	*LIPC*	CAD	0.76	0.12	0.32
		IS	0.07	0.74	0.39
		CVD	0.31	0.25	0.68
rs17231506	*CETP*	CAD	0.29	0.41	0.84
		IS	0.91	0.84	0.25
		CVD	0.41	0.36	0.41
rs9953437	*LIPG*	CAD	0.97	0.96	0.41
		IS	0.64	0.77	0.41
		CVD	0.88	0.89	0.26

Abbreviations. SNP, single nucleotide polymorphism; CAD, coronary artery disease; IS, ischemic stroke; CVD, cardiovascular disease. Note: ^a^ The interaction *p*-values for SNP × macronutrients (protein, carbohydrate, and fat) were obtained using a general linear model in SPSS (IBM SPSS statistics version 30.0.0), adjusted for age, gender, smoking status, drinking status, regular exercise, BMI, and total energy intake (kcal). ^b^ Protein (%) intake groups: high (≥15%) and low (<15%); ^c^ carbohydrate (%) intake groups: high (≥65%) and low (<65%); ^d^ fat (%) intake groups: high (≥20%) and low (<20%).

## Data Availability

Data analyzed in this study were obtained from the Korean Genome and Epidemiology Study and are available at the following website: https://coda.nih.go.kr/usab/koges/intro.do (accessed on 1 December 2024).

## Abbreviations

The following abbreviations are used in this manuscript:

CVD	Cardiovascular diseases
GWAS	Genome-wide association studies
SNPs	Single nucleotide polymorphisms
HDL	High-density lipoprotein
KoGES	Korean Genome and Epidemiology Study
DRIs	Dietary Reference Intakes
AMDR	Acceptable Macronutrient Distribution Ranges
TEI	Total energy intake
GLM	Generalized linear models
CAD	Coronary artery disease
RCT	Reverse cholesterol transport
LPL	Lipoprotein lipase
PPARs	Peroxisome proliferator-activated receptors
